# Decoding the Relationship Between Polycystic Ovary Syndrome and Hormonal Dependencies of Anti-Müllerian Hormone and Other Markers

**DOI:** 10.3390/biomedicines13061341

**Published:** 2025-05-30

**Authors:** Dominika Gałczyńska, Jakub Daniluk, Aleksandra Buczek-Kutermak, Paweł Pruś, Dagmara Pluta

**Affiliations:** Department of Gynecological Endocrinology, Faculty of Medical Sciences in Katowice, Medical University of Silesia in Katowice, 40-055 Katowice, Poland

**Keywords:** polycystic ovary syndrome, anti-Müllerian hormone, hyperandrogenism, body mass index, testosterone, PCOS phenotypes

## Abstract

**Background:** The objective of this study was to evaluate the influence of polycystic ovary syndrome (PCOS) on various laboratory measurements, especially hormonal and metabolic parameters, as well as clinical measurements including hirsutism and acne assessment, with consideration of different PCOS phenotypes. This study was focused mainly on the correlation between anti-Müllerian hormone (AMH) levels and other hormonal measurements. **Methods:** This single-center retrospective study included 296 patients with diagnosed PCOS according to the Rotterdam criteria. All participants of this study underwent blood tests between the 2nd and the 6th day of their menstrual cycle. **Results:** In statistical analysis, a strong significant correlation of AMH with androstenedione (r = 0.48, *p* < 0.0001), luteinizing hormone (LH) (r = 0.45, *p* < 0.0001), total testosterone (r = 0.34, *p* < 0.0001), 17-hydroxyprogesterone (r = 0.31, *p* < 0.0001), and cortisol after dexamethasone (r = 0.15, *p* = 0.011) was observed. In addition, significant negative correlations were found with follicle-stimulating hormone (FSH) (r = −0.21, *p* < 0.0001), weight (r = −0.15, *p* = 0.010), glucose 0′ (r = −0.14, *p* = 0.014), hip circumference (r = −0.14, *p* = 0.017), and body mass index (BMI) (r = −0.14, *p* = 0.018). A weak correlation with waist circumference of *p* = 0.06 was also observed. **Conclusions:** AMH serum levels showed a positive correlation with hyperandrogenism and a negative correlation with metabolic factors, although its relationship with BMI is more complex. There were no significant differences in AMH levels across the four PCOS phenotypes or when categorized into hyperandrogenic and normoandrogenic subtypes.

## 1. Introduction

Polycystic ovary syndrome is a prevalent endocrine disorder affecting approximately 10–13% of women of reproductive age [1]. PCOS is characterized by a constellation of symptoms including hyperandrogenism, oligo-anovulation, and polycystic ovarian morphology. It has profound implications for a woman’s reproductive, metabolic, and psychological health, often leading to complications such as infertility, type 2 diabetes, and increased cardiovascular risk. The exact cause of PCOS is not fully understood, but it is believed to involve a combination of genetic, hormonal, and environmental factors.

Although no specific genes were identified to be responsible for PCOS, family predisposition, especially among first-degree relatives, is proven to be a risk factor. Almost 40% of sisters of PCOS patients are also diagnosed with this disorder [2].

Hormonal background includes the pituitary being overstimulated with frequent gonadotropin-releasing hormone (GnRH) pulses, causing increased LH secretion and androgen synthesis defect in both ovaries and adrenal glands. Due to pituitary overstimulation by GnRH, the LH level is increased. An increased LH level stimulates ovarian steroidogenesis, causing increased synthesis of androgens. Hyperinsulinemia, through synergistic effect with LH, intensifies this process by directly stimulating ovarian granulosa and theca cells, and indirectly by insulin-like growth factor-1 (IGF-1). Hyperinsulinemia increases activity of hypothalamus–pituitary–adrenal axis, causing adrenocorticotropine hormone (ACTH)-dependent secretion of adrenal androgens. At the same time, it directly sensitizes ovarian granulosa cells to LH and causes an increased number of LH and IGF receptors, which induces ovarian steroidogenesis. An increased insulin level inhibits hepatic sex hormone-binding globulin (SHBG) synthesis, which leads to an increased level of bioavailable testosterone [3].

In PCOS patients’ blood, C-reactive protein (CRP), inflammatory cytokines like interleukin 1 (Il-1), interleukin 6 (Il-6), and tumor necrosis factor α (TNF-α) levels are elevated. TNF-α and Il-6 levels are also elevated in ovarian follicular fluid. One of the mechanisms of chronic inflammation in PCOS is adipocyte hypertrophy, which causes adipose tissue hypoperfusion and hypoxia. This process stimulates the activation of transcription factor- nuclear factor kappa-light-chain-enhancer of activated B cells (NFκB) in circulating mononuclear cells, which indicates proinflammatory cytokine production [4].

Early diagnosis, personalized treatment, and comprehensive management strategies are essential to address the reproductive, metabolic, and psychological aspects of the condition [5,6].

An anti-Müllerian hormone is produced by the granulosa cells of ovarian follicles and is considered a marker of ovarian reserve and also a regulator of folliculogenesis by inhibiting the initial recruitment of primordial follicles and reducing follicle sensitivity to FSH.

In women with PCOS, AMH levels are typically elevated due to the increased number of small antral follicles. The AMH can be a useful adjunctive marker in diagnosing PCOS, particularly when ultrasound features are ambiguous or when ovarian morphology is difficult to assess. Elevated AMH levels in PCOS reflect the disrupted folliculogenesis, causing anovulatory cycles, and the underlying pathomechanism might be a chronic inflammation process, characteristic of this syndrome. The AMH is upregulated by androgen levels and has the ability to suppress aromatase activity, causing hyperandrogenism [2].

Understanding AMH’s role in PCOS provides potential therapeutic avenues. Treatments that modulate AMH levels or their effects on folliculogenesis could improve ovulatory function and reduce hyperandrogenic symptoms [7,8].

Our focus in this study was to explore AMH levels in our study group, which consisted of Caucasian women with diagnosed PCOS, the correlation between the AMH and other hormonal and metabolic parameters we measured, and whether it varies in terms of PCOS phenotypes.

## 2. Materials and Methods

Our study involved 296 newly diagnosed Polish women with polycystic ovary syndrome, who were hospitalized in the Department of Endocrinological Gynecology between December 2021 and September 2022. The inclusion criteria were women aged 18–40 with confirmed PCOS diagnosis based on the Rotterdam criteria. According to the Rotterdam criteria, a diagnosis of PCOS requires the presence of at least two of the following three features: oligo- or anovulation (irregular cycles < 21 or >35 days), hyperandrogenism (clinical or biochemical: above the normal range for total testosterone, free testosterone, dehydrepiandrosterone sulfate, or androstenedione), and polycystic ovaries (≥12 follicles measuring 2–9 mm in diameter and/or an ovarian volume > 10 mL in at least one ovary). Patients were classified into different phenotypes. The Rotterdam criteria categorize PCOS into four distinct phenotypes based on the presence of oligo- or anovulation, hyperandrogenism, and polycystic ovaries. Phenotype A (classic PCOS)—oligo- or anovulation, hyperandrogenism, and polycystic ovaries; phenotype B (non-PCO PCOS)—oligo- or anovulation and hyperandrogenism, but normal ovarian morphology; phenotype C (ovulatory PCOS)—hyperandrogenism and polycystic ovaries, but regular ovulation; phenotype D (mild PCOS)—oligo- or anovulation and polycystic ovaries, but normal androgen levels. Excluded from the study were patients with hypothalamic-pituitary dysfunction of insufficiency, Cushing’s syndrome, acromegaly, hyperprolactinemia, decreased ovarian reserve, premature ovarian insufficiency, congenital adrenal hyperplasia and/or tumors, and thyroid insufficiency, including Hashimoto’s disease [5,9].

All participants of this study underwent blood tests between the 2nd and the 6th day of their menstrual cycle. On the first day of hospitalization, blood samples were drawn at 10.00 o’clock and the following hormones were measured: AMH, FSH, LH, prolactin (PRL), SHBG, free testosterone, total testosterone, androstenedione, dehydroepiandrosterone sulfate (DHEA-S), and 17-OH-progesterone. Next, patients fasted for 12 h, and the blood was drawn on the next morning (second day of hospitalization) for glucose, insulin, lipid profile, thyroid-stimulating hormone (TSH), free thyroxine (fT4), and liver enzymes. Afterward, oral glucose tolerance test (OGTT) was performed—glucose was measured at 0′ and 120′, with cut-off values accordingly: >99 mg/dL and >140 mg/dL, and HOMA-IR calculated with a cut-off point of 2.0. Moreover, on this day, the ultrasound screening took place. On the third day, we drew blood for cortisol, accordingly at 8.00 a.m. and 11 p.m.; later on, patients took 1 mg of dexamethasone. On the fourth day of hospitalization, we drew blood at 8.00 a.m. for the cortisol after dexamethasone. Venous blood samples were collected as presented above and sent to the hospital’s diagnostic laboratory every day.

The lipid profile and glucose levels were assessed using colorimetric methods with an AU 680 analyzer (Beckman Coulter, Brea, CA, USA). Additionally, serum concentrations of various hormones (such as estradiol, follicle-stimulating hormone, luteinizing hormone, total and free testosterone, 17-OH-progesterone, androstenedione, cortisol, dehydroepiandrosterone sulfate, sex hormone-binding globulin, and insulin) were measured using chemiluminescence with reagents from Abbott (Architect i2000SR; Chicago, IL, USA). Ultrasound examinations were conducted using the Voluson 730 Expert, and anthropometric measurements (body mass and height) were taken.

For clinical assessment of hyperandrogenism, the most widely used method is assessing hirsutism in the Ferriman–Gallwey Score, which involves scoring hair growth in 9 body areas (upper lip, chin, chest, upper and lower back, upper and lower abdomen, arms, and thighs). Each area is scored from 0 (no hair) to 4 (extensive hair growth). A total score of 8 or higher typically indicates hirsutism; 8 to 16 points represents mild hirsutism, 17–26 points, moderate, and 27–32 points, severe hirsutism. Whereas acne is assessed with the Acne Severity Index, which evaluates the number and type of acne lesions on the face, chest, and back. Severity is categorized as mild, moderate, or severe based on the count and type of lesions [10].

Qualitative data are presented as the number of cases with the percentage and its 95% confidence interval. Quantitative variables were analyzed using a graphical method based on quantile–quantile plots (QQ-plot) and histograms. To compare the differences for normally distributed variables, the Student’s *t*-test was used, while for non-normal distribution variables—the U-Mann–Whitney test. In order to compare more than one group, a nonparametric analysis of variance was performed—Kruskal–Wallis with the post hoc test of multiple comparisons. The correlation analysis was performed using the Spearman rank correlation coefficient for non-normal distributed variables and the Pearson correlation coefficient for normally distributed variables. For the purpose of reducing the number of dimensions in the data set, factor analysis using the principal components method, using the scree diagram, was used to select the number of components used. The analysis was performed in R in the RStudio environment (Posit team (2024). Posit Software, PBC, Boston, MA; R: A language and environment for statistical computing. R Foundation for Statistical Computing, Vienna, Austria.). *p*-values less than 0.05 were considered significant.

All methods were carried out in accordance with relevant guidelines and regulations approved by the Department of Gynecological Endocrinology, Medical University of Silesia. Informed consent was waived and obtained from all hospitalized patients and/or their legal guardians.

## 3. Results

Table 1 presents all quantitative variables that were measured in our patients during hospitalization. We collected the data that included hormonal panel measurements, as well as metabolic parameters that were essential for this study. Table 1 sums up the statistical characteristics of all collected data by presenting minimum, maximum, median, and average values, quartiles, standard deviation, and confidence interval of 95%. Qualitative data contained PCOS phenotypes, glucose metabolism disturbances, acne, and hirsutism. In the group of hospitalized patients, 51.01% presented fully developed PCOS-phenotype A, 13.51% phenotype B, 17.91% phenotype C, and 17.57% phenotype D. In the hospitalized PCOS patients, 49 (17%) women suffered from glucose metabolism disturbances—impaired glucose tolerance and impaired fasting glucose. In 35.47% of patients, no acne was observed, whereas 49.67% faced mild-to-moderate lesions, and 2.7% suffered from severe and very severe forms of acne. Hirsutism occurred in about half of the patients (53.38%), mostly in mild severity (35.81%) (Table 2).

The group of PCOS patients was also divided into hyperandrogenic vs. normoandrogenic groups. Table 3 presents a comparison between the mentioned groups of patients.

According to the statistical analysis of our study group, only the fT4 measurement has reached a *p*-value below 0.05. The result described above shows that hyperandrogenism can interfere with fT4 measurement, and due to this interference, measured values can be higher. Other parameters like TSH or cortisol concentration at 8:00 AM were close to reaching statistical importance with *p*-values = 0.069 and 0.096, accordingly.

### 3.1. Correlation with Serum AMH Concentrations and Patients’ Hormonal and Metabolic Parameters

A strong significant correlation of AMH with androstenedione (r = 0.48, *p* < 0.0001), LH (r = 0.45, *p* < 0.0001), total testosterone (r = 0.34, *p* < 0.0001), 17-hydroxyprogesterone (r = 0.31, *p* < 0.0001), and cortisol after dexamethasone (r = 0.15, *p* = 0.011) was observed. These correlations were positive. In addition, significant negative correlations were found with FSH (r = −0.21, *p* < 0.0001), weight (r = −0.15, *p* = 0.010), glucose 0′ (r = −0.14, *p* = 0.014), hip circumference (r = −0.14, *p* = 0.017), and BMI (r = −0.14, *p* = 0.018). A weak correlation with waist circumference of *p* = 0.06 was also observed.

**Figure 1:**
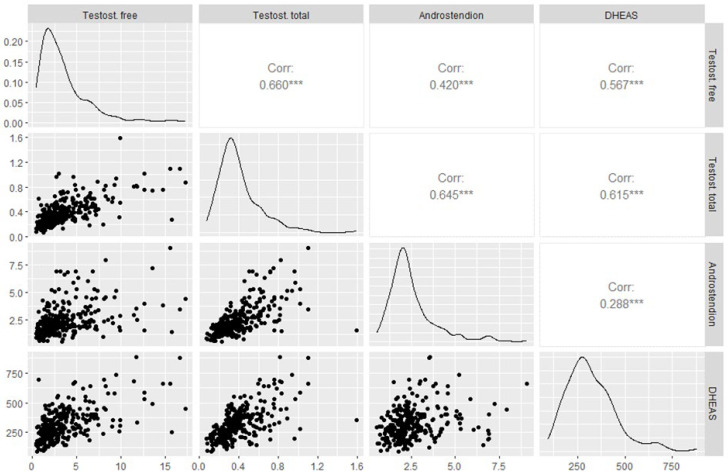
Analysis of correlations between androgen hormone concentrations. *** for partial correlations.

**Figure 2:**
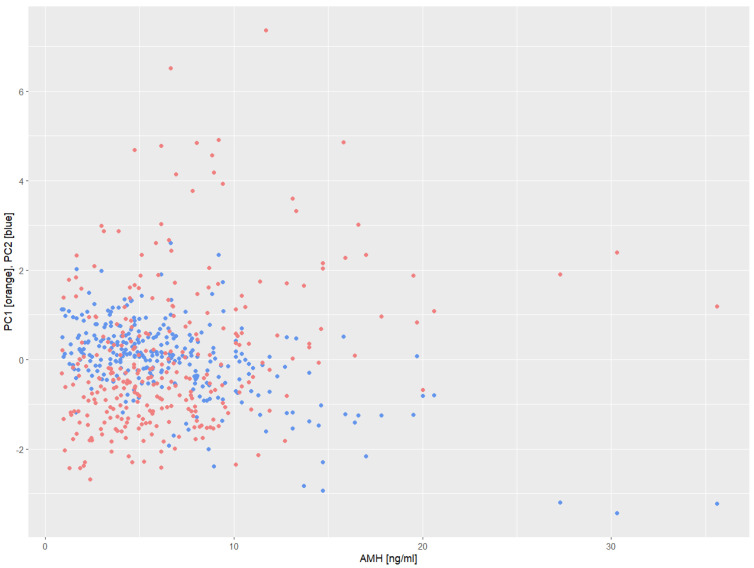
Correlation between serum AMH concentration and factor 1 and factor 2.

Due to strong correlations between hormone concentrations (Figure 1), it was decided to perform a factor analysis using the principal components method. The principal components analysis isolated two factors, with summing explaining 83.95% of the variance of the system. Factor 1 explained 65.42%, while factor 2 explained 18.53%. It was observed that factor 1 was positively correlated with all components of the analysis (PC1 with free testosterone, total testosterone, androstenedione, and DHEA-S consequently 0.511; 0.565; 0.442; 0.473), while factor 2 was strongly negatively correlated with androstenedione (PC2 = −0.763) concentration and positively with DHEA-S (PC2 = 0.587). Its correlations with testosterone were weak (free testosterone PC2 = 0.244, total testosterone −0.115). AMH appears to be positively associated with androgen hormones. Androstenedione appears to have a clear effect on the increase in AMH concentration, while an increase in DHEA-S contributes to a decrease in AMH concentration, which was not observed in simple univariate analyses (Figure 2).

### 3.2. Comparison of AMH by PCOS Phenotype

**Figure 3:**
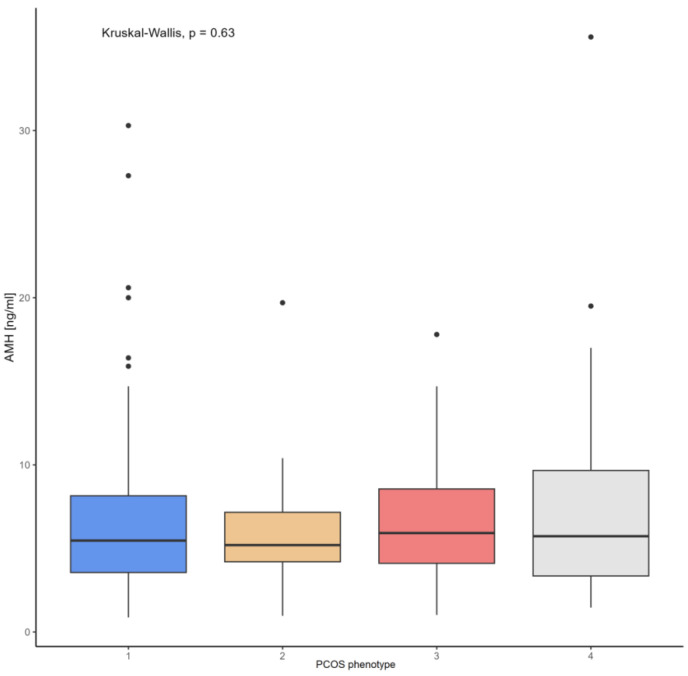
Comparison of AMH concentrations and PCOS phenotype.

**Figure 4:**
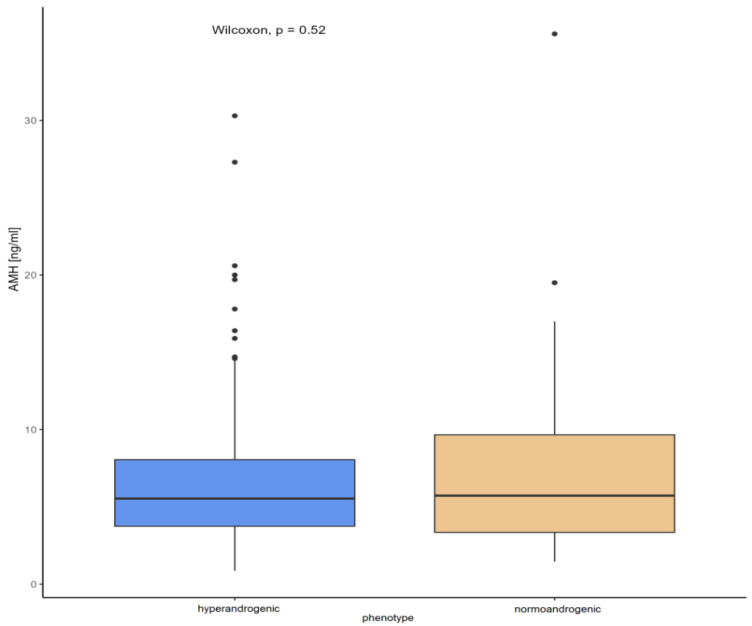
Comparison of AMH concentrations and hyper- vs. normoandrogenic phenotype.

According to the Kruskal–Wallis test, there were no significant differences between AMH concentration and the four PCOS phenotypes (Figure 3), nor when PCOS was divided into hyper- and normoandrogenic phenotypes (Figure 4).

### 3.3. Serum AMH Concentrations and Body Mass Index

**Figure 5:**
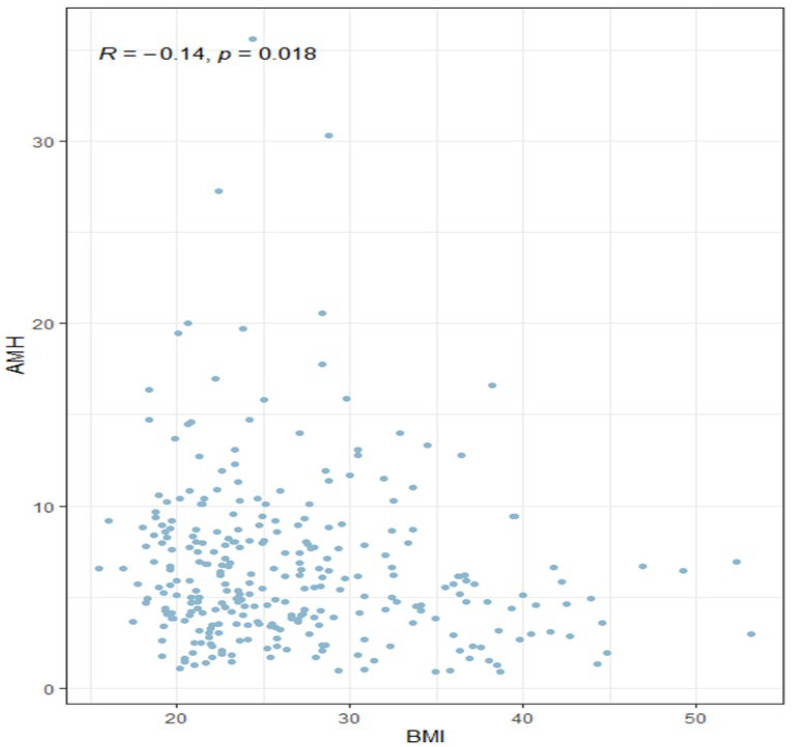
Correlation between BMI and serum AMH concentrations (Spearman’s correlation coefficient).

A significant difference was observed between AMH levels among underweight patients and obese patients, where the average AMH concentration was lower. Also, a significant difference between normal-weight and obese patients was presented. An additional correlation analysis showed a negative relationship between AMH concentration and BMI. (Figure 5).

## 4. Discussion

In this study, almost 20% of women diagnosed with PCOS presented glucose metabolism disorders, which is typical for the syndrome, and has already been established in previous studies. According to Nayak B S et al., women with PCOS had higher levels of fasting glucose independently, whether they were obese or non-obese [11]. Our study shows a significant negative correlation between AMH serum levels and glucose 0′ in OGTT, weight, hip circumference, and BMI (r = −0.14, *p* = 0.014; r = −0.15, *p* = 0.010; r = −0.14, *p* = 0.017; r = −0.14, *p* = 0.018, respectively), which is consistent with data already described in the past [12,13]. Impaired glucose tolerance and impaired fasting glucose are prediabetes states that are independent risk factors associated with type 2 diabetes. Performing an oral glucose tolerance test in every PCOS patient allows prediabetes to be diagnosed, implying that a lifestyle intervention is required to revert glucose levels to a proper range [14,15]. Maintaining normal glucose levels is associated with maintaining proper body weight and BMI. Patients with PCOS facing a metabolic component, including obesity, present with decreasing AMH levels [16].

Women suffering from PCOS have a positive correlation between serum AMH concentrations and LH, which has already been proven by researchers [7,17,18]. Therefore, it points out an interaction between AMH, LH, and serum androgen levels. This suggests that the one of underlying pathogenesis factors of PCOS is strictly connected to the hypothalamus–pituitary–ovary axis, where LH secretion by the anterior pituitary is increased due to disturbed gonadotropin-releasing hormone pulses. Moreover, decreased FSH levels, affecting the LH/FSH ratio, result in elevated androgen synthesis in theca cells, leading to excess androgen production in the end. Furthermore, the mentioned mechanisms contribute to follicular development holdup, causing accumulation of antral follicles, and a PCOM and AMH level increase [18]. In our study, results showed a significant positive correlation between the level of AMH, LH, and androgens, specifically total testosterone (r = 0.34, *p* < 0.0001) and 17-hydroxyprogesterone (r = 0.31, *p* < 0.0001), which proves the mentioned connection of pathomechanisms in our PCOS patients. Further analysis emphasized that androstenedione and AMH clearly influence each other. Pomme I. H. G. Simons et al. and Pascal Pigny have similar conclusions about androstenedione and AMH being in a tight significant association [19]. The underlying cause might be the androstenedione level upregulating AMH gene expression through the androgen receptor pathway in granulosa cells. Further, another cause is that androstenedione suppresses aromatase activity, causing a decrease in androgen-to-estrogen conversion, resulting in reduced estrogen-mediated feedback, contributing to hormonal imbalance and maintaining elevated AMH levels.

According to our analysis, higher levels of DHEA-S might lead to lower AMH serum concentrations, but this was only proven by performing factor analysis using the principal components method, not simple univariate analysis. Hélène Boucher et al., in their latest study, did not find a correlation between AMH and DHEA-S [20]. The hypothesis behind this phenomenon was that DHEA-S higher levels are due to the stimulation of steroidogenesis in adrenal glands, and this is unlikely in ovaries, so there is no direct link between AMH levels and DHEA-S excessive production.

This topic needs further investigation and analysis in our group of patients to draw any specific conclusions about the correlation between DHEA-S and AMH levels.

Our results presented significant negative correlations between FSH (r = −0.21, *p* < 0.0001) and AMH concentration in our PCOS hospitalized patients. Serum levels of FSH in PCOS women are reported to vary from low to normal ranges. In accordance with previous studies, a negative relationship between serum AMH and FSH levels has been established, but the underlying mechanism still remains unclear [21]. The literature provides a theory of accelerated pulse frequency of GnRH and increased AMH concentration leading to increased pituitary LH secretion, resulting in FSH secretion disturbance [22], which was confirmed in our results.

Patients with PCOS have 2–4 times higher levels of AMH compared to healthy women. This phenomenon is explained by the pathomechanism of AMH secretion by preantral and small antral follicles, where the amount is higher due to the disease itself. In vitro cell studies have shown 75-fold higher AMH secretion in anovulatory patients with PCOS and 20-fold higher hormone levels in PCOS ovulating patients than in healthy women [7]. This leads to the conclusion that the mechanism of excessive AMH production is related to hyperandrogenism, although it is not fully explained. The mechanism of insulin resistance is present in 75% of lean and 95% of overweight and obese PCOS patients who suffer from menstrual disorders and hyperandrogenism. Hyperinsulinemia reduces the production of SHBG in the liver, thus leading to increased levels of free testosterone in the blood. Moreover, it stimulates the overproduction of androgens in the theca cells. Studies carried out by N Dokuzeylül Güngör et al. and Enrico Carmina et al. presented that the higher the BMI level, the lower the concentration of AMH in women with PCOS [22,23]. Our study also indicated that as BMI increased, the average concentration of AMH in the blood decreased (Figure 5). On the other hand, the AMH level in underweight patients was also significantly lower than in patients with normal BMI, which draws attention to the necessity of exploring this subject.

To the strengths of this study, we can include a highly selected inclusion-criteria homogenic study group, a wide variety of analyzed clinical and laboratory variables, and a highly standardized setup of clinical and laboratory assessments, which minimizes confounding factors. To the limitations of this research, we can include the absence of a control group, and a low number of patients with a normoandrogenic phenotype compared to the hyperandrogenic phenotype, which results in underrepresentation of the aforementioned group. Therefore, we will seek to remove limitations as much as possible in future research on this topic. Future directions of the study might include a long-term follow-up of the participants of the study, which would show the evolution of hormonal and metabolic parameters, including AMH, throughout the years, enable comparison, and perhaps lead to new discoveries and directions of AMH’s role in PCOS. The AMH level in underweight patients was significantly lower than in patients with normal BMI, which draws attention to the necessity of exploring this subject. Also, the topic of correlation between D-HEAS and AMH levels might be a proper direction to investigate in the future.

## 5. Conclusions

In conclusion, AMH serum levels were positively correlated with hyperandrogenism and negatively with metabolic factors, although the connection between AMH and BMI has a more complex nature. There were no significant differences between AMH and four PCOS phenotypes, when divided into either hyper- or normoandrogenic subtypes.

AMH continues to be a diagnostic challenge in gynecological endocrinology, including patients suffering from PCOS, and warrants ongoing investigation and a deeper understanding of its role.

## Figures and Tables

**Table 1:** General characteristics of quantitative variables *.

Variable	min	max	Median	q1	q3	Average	SD	95% CI	
17-OH-P (nmol/L)	0.08	3.67	0.965	0.68	1.3	1.027	0.514	0.968	1.086
AMH (ng/mL)	0.87	35.6	5.62	3.64	8.292	6.583	4.546	6.063	7.103
Androstenedione (ng/mL)	0.55	9.07	2.17	1.74	2.9	2.517	1.346	2.363	2.671
BMI (kg/m^2^)	15.519	53.15	24.675	21.404	29.805	26.643	6.95	25.848	27.438
Cholesterol (mmol/L)	101	258	163.5	143	181	164.165	29.235	160.821	167.509
DHEA-S (mcg/dL)	92	882	297	230.75	393	321.918	131.663	306.857	336.979
FSH (IU/L)	1.22	12.8	5.985	5.068	7.105	6.183	1.648	5.994	6.372
Glucose in 0′ (mg/dL)	72.5	129	84.6	81.15	88.35	85.115	6.503	84.37	85.86
Glucose in 120′ (mg/dL)	0	199	107	90.65	127	110.258	28.425	107.001	113.515
HDL (mmol/L)	21.3	113	55	45.475	67.275	56.967	15.61	55.181	58.753
HOMA-IR	0	15.678	1.67	1.148	2.51	2.182	1.814	1.975	2.389
Height (cm)	151	185	166	162	170	166.091	5.853	165.421	166.761
Hip (cm)	76	166	102	95	110	104.624	13.554	103.071	106.177
Insulin 0′ (µU/mL)	1.5	83	7.93	5.47	12.05	10.336	8.533	9.358	11.314
Cortisol at 23:00 (µg/dL)	0.52	13.4	2.005	1.317	3.463	2.746	2.116	2.504	2.988
Cortisol at 8:00 (µg/dL)	2.74	26.4	12.55	9.945	15.225	12.736	3.79	12.302	13.17
Cortisol after dexamethasone (µg/dL)	0.26	2.12	0.56	0.48	0.69	0.598	0.193	0.576	0.62
LDL (mmol/L)	28.4	179.9	85.4	69.29	104.56	88.325	27.016	85.229	91.421
LH (IU/L)	1.73	506	6.95	4.928	9.762	9.908	29.346	6.551	13.265
PRL 10:00 (ng/mL)	3.37	57.4	10.2	8.01	13.4	11.338	5.264	10.736	11.94
SHBG (nmol/L)	6.42	197	44.95	30.075	63.225	51.297	31.134	47.736	54.858
TSH (µIU/mL)	0.414	7.3	1.745	1.23	2.3	1.857	0.859	1.759	1.955
Testost. Free (ng/mL)	0.38	17.3	2.74	1.615	4.55	3.585	2.938	3.249	3.921
Testost. Total (ng/mL)	0.07	4.43	0.347	0.263	0.481	0.411	0.313	0.375	0.447
Triglycerides (mmol/L)	34.9	402	83.95	62.275	111.25	94.936	47.883	89.459	100.413
WHR	0.56	1.11	0.79	0.745	0.865	0.813	0.092	0.802	0.824
Waist (cm)	57	143	81	72	94	84.889	16.505	83.001	86.777
Weight (kg)	39	150	67.6	59	84	73.674	20.322	71.349	75.999
fT4 (pmol/L)	0.89	4.72	1.2	1.11	1.3	1.219	0.249	1.19	1.248

* Legend: SD—standard deviation, 17-OH-P—17-hydroxyprogesterone, AMH—anti-Müllerian, BMI—body mass index, DHEA-S—dehydroepiandrosterone sulfate, FSH—follicle-stimulating hormone, HDL—high-density lipoprotein, HOMA-IR—Homeostatic Model Assessment of Insulin Resistance, LDL—low-density lipoprotein, LH—luteinizing hormone, PRL—prolactin, SHBG—sex-hormone binding globulin, TSH—thyroid-stimulating hormone; WHR—waist–hip ratio; fT4—free thyroxine.

**Table 2:** General characteristics of qualitative variables.

	n	%	95% CI	
**Glucose metabolism disturbances**				
no	247	83.45%	79.21%	87.68%
yes	49	16.55%	12.32%	20.79%
**Acne**				
clear	105	35.47%	30.02%	40.92%
almost clear	90	30.41%	25.16%	35.65%
mild	57	19.26%	14.76%	23.75%
moderate	36	12.16%	8.44%	15.89%
severe	7	2.36%	0.63%	4.10%
very severe	1	0.34%	0.00%	1.00%
**PCOS phenotype**				
hyperandrogenic A	151	51.01%	45.32%	56.71%
hyperandrogenic B	40	13.51%	9.62%	17.41%
hyperandrogenic C	53	17.91%	13.54%	22.27%
Normoandrogenic	52	17.57%	13.23%	21.90%
**Hirsutism**				
no hirsutism	158	53.38%	47.70%	59.06%
mild hirsutism	106	35.81%	30.35%	41.27%
moderate hirsutism	29	9.80%	6.41%	13.18%
severe hirsutism	3	1.01%	0.00%	2.15%

**Table 3:** Hyperandrogenic vs. normoandrogenic PCOS patients **.

	Hyperandrogenic	Normoandrogenic	
**n**	**244**	**52**	***p***
Weight (kg) (median [IQR])	68.00 [59.00, 84.00]	67.10 [57.75, 85.25]	0.849
Height (cm) (mean (SD))	166.15 (5.70)	165.82 (6.57)	0.711
BMI (kg/m^2^) (median [IQR])	24.61 [21.25, 29.71]	24.95 [22.18, 30.46]	0.415
Waist (cm) (median [IQR])	82.00 [72.00, 94.00]	80.00 [71.75, 96.25]	0.725
Hip (cm) (median [IQR])	102.00 [94.50, 110.00]	102.00 [97.00, 112.00]	0.671
WHR (mean (SD))	0.82 (0.09)	0.80 (0.10)	0.319
Triglycerides (mmol/L) (median [IQR])	83.40 [61.38, 111.00]	89.00 [68.10, 114.50]	0.493
Cholesterol (mmol/L) (mean (SD))	165.07 (29.96)	159.94 (25.37)	0.252
LDL (mmol/L) (mean (SD))	88.77 (27.45)	86.25 (25.04)	0.542
HDL (mmol/L) (mean (SD))	57.40 (15.90)	54.93 (14.14)	0.301
17-OH-P (nmol/L) (median [IQR])	0.93 [0.69, 1.30]	0.98 [0.66, 1.24]	0.947
Testost, free (ng/mL) (median [IQR])	2.78 [1.62, 4.44]	2.40 [1.55, 4.76]	0.935
Testost, total (ng/mL) (median [IQR])	0.35 [0.26, 0.48]	0.35 [0.28, 0.50]	0.762
Androstenedione (ng/mL) (median [IQR])	2.14 [1.70, 2.89]	2.22 [1.78, 2.96]	0.485
DHEA-S (mcg/dL) (median [IQR])	300.00 [233.50, 390.25]	287.00 [229.25, 400.25]	0.918
FSH (IU/L) (median [IQR])	6.06 [5.07, 7.16]	5.60 [5.00, 6.76]	0.318
LH (IU/L) (median [IQR])	6.96 [4.92, 9.76]	6.82 [5.03, 9.16]	0.731
**fT4 (pmol/L) (median [IQR])**	**1.20 [1.14, 1.30]**	**1.12 [1.09, 1.23]**	**0.018**
TSH (µIU/mL) (median [IQR])	1.78 [1.25, 2.34]	1.57 [1.15, 2.02]	0.069
SHBG (nmol/L) (median [IQR])	45.10 [30.17, 63.23]	40.95 [29.98, 62.83]	0.894
PRL (ng/mL) 10:00 (median [IQR])	10.20 [7.95, 13.90]	9.68 [8.41, 12.35]	0.601
AMH (ng/mL) (median [IQR])	5.54 [3.75, 8.05]	5.73 [3.35, 9.66]	0.515
Insulin 0′ (µU/mL) (median [IQR])	7.92 [5.46, 11.95]	8.12 [5.75, 12.17]	0.581
Cortisol 8:00 (µg/dL) (median [IQR])	12.40 [9.82, 14.93]	13.50 [10.75, 15.75]	0.096
Cortisol 23:00 (µg/dL) (median [IQR])	1.97 [1.32, 3.63]	2.17 [1.29, 3.22]	0.877
Cortisol after dexamethasone (µg/dL) (median [IQR])	0.55 [0.48, 0.69]	0.60 [0.49, 0.68]	0.499
Glucose 0′ (mg/dL) (median [IQR])	84.60 [81.15, 88.55]	83.65 [81.45, 87.78]	0.798
Glucose 120′ (mg/dL) (median [IQR])	106.00 [90.15, 127.00]	110.00 [93.97, 129.50]	0.732
HOMA-IR (median [IQR])	1.67 [1.14, 2.51]	1.69 [1.26, 2.53]	0.583

** Legend: IQR—interquartile range, SD—standard deviation, SE—standard error, 17-OH-P—17-hydroxyprogesterone, AMH—anti-Müllerian, BMI—body mass index, DHEA-S—dehydroepiandrosterone sulfate, FSH—follicle-stimulating hormone, HDL—high-density lipoprotein; HOMA-IR—Homeostatic Model Assessment of Insulin Resistance, LDL—low-density lipoprotein, LH—luteinizing hormone, PRL—prolactin, SHBG—sex-hormone binding globulin, TSH—thyroid-stimulating hormone; WHR—waist–hip ratio; **fT4—free thyroxine**-statistically significant.

## Data Availability

The data sets generated during and/or analyzed during the current study are available in the Department of Gynecological Endocrinology, Faculty of Medical Sciences in Katowice, Medical University of Silesia in Katowice, Katowice, Poland. In the case of data requests, we kindly ask that Dominika Gałczyńska, MD, be contacted.
